# Special Issue: 2D Layered Nanomaterials and Heterostructures for Electronics, Optoelectronics, and Sensing

**DOI:** 10.3390/nano15110851

**Published:** 2025-06-02

**Authors:** Filippo Giannazzo, Federica Bondino, Luca Seravalli, Simonpietro Agnello

**Affiliations:** 1 Consiglio Nazionale della Ricerche (CNR), Istituto per la Microelettronica e Microsistemi (IMM), Strada VIII, 5, 95121 Catania, Italy; 2 Consiglio Nazionale delle Ricerche (CNR), Istituto Officina dei Materiali (IOM), Area Science Park, S.S. 14 Km. 163, 5, Basovizza, 34149 Trieste, Italy; bondino@iom.cnr.it; 3 Consiglio Nazionale delle Ricerche (CNR), Istituto dei Materiali per l’Elettronica ed il Magnetismo (IMEM), Parco Area delle Scienze 37/a, 43124 Parma, Italy; luca.seravalli@imem.cnr.it; 4 Department of Physics and Chemistry Emilio Segré, University of Palermo, Via Archirafi 36, 90143 Palermo, Italy; simonpietro.agnello@unipa.it

Since the first report in 2004 on the electronic properties of graphene exfoliated from graphite [1], the research on two-dimensional materials (2DMs) has grown steadily. To date, hundreds of 2DMs have been investigated experimentally, whereas a database of up to 6000 monolayer structures has recently been compiled through computational studies [2]. Interestingly, the 2DM family covers the entire range of electronic properties, from semimetallic graphene and semiconducting transition metal dichalcogenides (TMDs) to insulating hexagonal boron nitride. Furthermore, van der Waals (vdW) heterostructures with engineered bandgaps have been realized by stacking different layers [3], enabling the demonstration of novel device concepts for electronics/optoelectronic, sensing, quantum, and energy applications.

In this context, this Special Issue, entitled “2D Layered Nanomaterials and Heterostructures for Electronics, Optoelectronics and Sensing”, features insightful contributions on the scalable growth of 2DMs, fabrication approaches of vdW heterostructures, advanced characterization methods (optical, vibrational, chemical, electrical), and the theoretical modeling of these heterostructures. Furthermore, it aims to address challenges involved in 2DM integration and device fabrication.

Specifically, the Special Issue includes 10 high-quality, original research papers covering the following topics:

i. Scalable synthesis of molybdenum disulfide (MoS_2_) through an advanced chemical vapor deposition (CVD) approach based on liquid Mo precursors [4], as well as by pulsed laser deposition (PLD) on substrates of interest for microelectronics (sapphire, gallium nitride) [5];
ii. First-principles calculations of electronic and optical properties of graphene, borophene, and boron carbide 2D heterostructures [6];
iii. Advanced optical and electronic transport characterization of novel vdW heterostructures (Ta_2_NiS_5_/CrOCl) [7];
iv. Electronic and optoelectronic devices based on 2DM heterostructures, i.e., novel 2D field effect transistors with a tungsten diselenide (WSe_2_) channel and multilayer palladium diselenide (PdSe_2_) vdW contacts [8]; bipolar transistors based on a MoS_2_/WSe_2_/MoS_2_ heterostructure [9]; photo-transistors and self-powered photodetectors based on graphene/Si [10]; NiO/graphene/Si junctions [11]; photovoltaic devices [12]; and biosensors based on MoS_2_/WTe_2_ Schottky barriers [13].

This Special Issue spotlights relevant examples of ongoing research directions in the continuously expanding field of 2D materials and aims to serve as inspiration for researchers working in these areas.
